# Safety of dihydroartemisinin-piperaquine versus artemether-lumefantrine for the treatment of uncomplicated *Plasmodium falciparum* malaria among children in Africa: a systematic review and meta-analysis of randomized control trials

**DOI:** 10.1186/s12936-021-04032-2

**Published:** 2022-01-04

**Authors:** Dawit Getachew Assefa, Eden Dagnachew Zeleke, Wondwosen Molla, Nebiyu Mengistu, Ahmedin Sefa, Andualem Mebratu, Asresu Feleke Bate, Etaferaw Bekele, Gizachew Yesmaw, Eyasu Makonnen

**Affiliations:** 1 Center for Innovative Drug Development and Therapeutic Trials for Africa (CDT-Africa), College of Health Sciences, Addis Ababa University, Addis Ababa, Ethiopia; 2 School of Public Health, College of Health Science and Medicine, Dilla University, Dilla, Ethiopia; 3 Department of Midwifery, College of Health Science, Bule Hora University, Bule Hora, Ethiopia; 4 Department of Midwifery, College of Health Science and Medicine, Dilla University, Dilla, Ethiopia; 5 Department of Psychiatry, College of Health Science and Medicine, Dilla University, Dilla, Ethiopia; 6 Department of Nursing, College of Health Science and Medicine, Dilla University, Dilla, Ethiopia; 7 Department of Pharmacology and Clinical Pharmacy, College of Health Sciences, Addis Ababa University, Addis Ababa, Ethiopia

**Keywords:** Uncomplicated *Plasmodium falciparum*, Adverse event, Pediatrics, Children, Safety, Randomized control trial, Artemisinin-based combination therapy, Dihydroartemisinin-piperaquine, Artemether-lumefantrine, Systematic review, Meta-analysis, Africa

## Abstract

**Background:**

The efficacies of artemisinin based combinations have been excellent in Africa, but also comprehensive evidence regarding their safety would be important. The aim of this review was to synthesize available evidence on the safety of dihydroartemisinin-piperaquine (DHA-PQ) compared to artemether-lumefantrine (AL) for the treatment of uncomplicated *Plasmodium falciparum* malaria among children in Africa.

**Methods:**

A systematic literature search was done to identify relevant articles from online databases PubMed/ MEDLINE, Embase, and Cochrane Center for Clinical Trial database (CENTRAL) for retrieving randomized control trials comparing safety of DHA-PQ and AL for treatment of uncomplicated *P. falciparum* malaria among children in Africa. The search was performed from August 2020 to 30 April 2021. Using Rev-Man software (V5.4.1), the extracted data from eligible studies were pooled as risk ratio (RR) with 95% confidence interval (CI).

**Results:**

In this review, 18 studies were included, which involved 10,498 participants were included. Compared to AL, DHA-PQ was associated with a slightly higher frequency of early vomiting (RR 2.26, 95% CI 1.46 to 3.50; participants = 7796; studies = 10; I^2^ = 0%, high quality of evidence), cough (RR 1.06, 95% CI 1.01 to 1.11; participants = 8013; studies = 13; I^2^ = 0%, high quality of evidence), and diarrhoea (RR 1.16, 95% CI 1.03 to 1.31; participants = 6841; studies = 11; I^2^ = 8%, high quality of evidence) were more frequent in DHA-PQ treatment arm.

**Conclusion:**

From this review, it can be concluded that early vomiting, diarrhoea, and cough were common were significantly more frequent in patients who were treated with the DHA-PQ than that of AL, and both drugs are well tolerated. More studies comparing AL with DHA-PQ are needed to determine the comparative safety of these drugs.

**Supplementary Information:**

The online version contains supplementary material available at 10.1186/s12936-021-04032-2.

## Background

Malaria is the major cause for vast majority of deaths among children under the age of five [1–3]. In 2019, an estimated 229 million cases were reported globally from 87 malaria endemic countries [3], of which 215 million cases were reported in the World Health Organization (WHO) African Region [3]. The risk of malaria infections among children aged under five years was higher in 2018, and *Plasmodium falciparum* parasite were responsible for an estimated 24 million malaria cases in African children [1].

All African counties, where *P. falciparum* malaria is endemic, have introduced the recommended artemisinin-based combination therapy (ACT) in the confirmed cases of *P. falciparum* malaria since 2004 [1]. The artemisinin component is active against the asexual stage of the parasite responsible for the disease, but also the sexual stages of the parasite involved in the transmission to mosquitoes. The partner drug with a longer half-life eliminates the residual parasite over several weeks post treatment [4]. Artemisinin and partner drugs protect each other to prevent resistance development [5–8].

The efficacies of artemisinin-based combinations have been excellent in Africa [9, 10]. Artemether-lumefantrine (AL) is one of the most commonly used combinations in sub-Saharan Africa. It is the first-line treatment for uncomplicated malaria in several countries [11, 12]. AL showed good safety and tolerability profile [10, 13, 14]. Hence, previous reviews reported mild or moderate severity adverse event of gastrointestinal and nervous systems in patients who were treated with AL [15] and prolongation of the QTc interval; pyrexia, early vomiting, and diarrhoea were common in patients treated with DHA-PQ [16].

In the majority of African countries, the first-line treatment for uncomplicated malaria is generally AL or AS/AQ, with DHA-PQ as a second-line treatment in many countries [11, 12]. Most of the previous studies have compared the efficacies of AL and other artemisinin-based combinations [17, 18], but also comprehensive evidence regarding their safety would be important. Given the wide range of ACT available for treatment the malaria and their potential adverse events (AEs), it is vital to compare their safety profiles. This systematic review and meta-analysis was, therefore, to synthesize available evidence on the safety of dihydroartemisinin-piperaquine compared to artemether-lumefantrine for the treatment of uncomplicated *P. falciparum* malaria among children in Africa.

## Methods

This protocol has been registered at the International Prospective Register of Systematic Reviews (PROSPERO) database, ID: CRD42020200337 [19]. The methods and findings of the review have been reported according to the preferred reporting items for systematic reviews and meta-analyses (PRISMA 2020) [20].

### Eligibility criteria

The PICOS format was used to identify eligible studies [21].

### Participants

Children having uncomplicated falciparum malaria residing in Africa, regardless of gender, were included.

### Interventions

A target dose (range) of 4 (2–10) mg/kg bw per day dihydroartemisinin and 18 (16–27) mg/kg bw per day piperaquine given once a day for 3 days for children weighing ≥ 25 kg. The target doses and ranges for children weighing < 25 kg are 4 (2.5–10) mg/kg bw per day dihydroartemisinin and 24 (20–32) mg/kg bw per day piperaquine once a day for 3 days.

### Comparator

The 1:6 fixed dose combination tablet consisting artemether (20 mg) and lumefantrine (120 mg).

The body weight-adjusted dosages used have been: 25–35 kg, 3 tablets per dose: 15 to 25 kg, 2 tablets per dose; and < 15 kg, 1 tablet.

The medication administered twice a day for three days (total six doses). The first two doses taken eight hours apart; the third dose is taken after 24 h the first and then every 12 h on days 2 and 3**.**

### Outcome measures

Adverse events including serious adverse events were also assessed. An adverse event (AE) was defined as any unfavourable, unintended sign, symptom, syndrome, or disease that develops or worsens with the use of a medicinal product, regardless of whether it is related to the actual medicinal product. A serious AE was defined as any untoward medical occurrence that at any dose; resulted in death; was life threatening; requiring hospitalization or prolongation of hospitalization; resulted in a persistent or significant disability or incapacity; or caused a congenital anomaly or birth defect [22].

### Studies

Randomized controlled trials conducted in Africa which compared the safety of DHA-PQ versus AL for the treatment of uncomplicated falciparum malaria in children, written in English, and published between 2004 to April 2021 were included.

### Electronic searches

A systematic literature search was done to identify relevant articles from online databases PubMed/ MEDLINE, Embase, and Cochrane Center for Clinical Trial database (CENTRAL). The search was limited to human trials, randomized control trials, and published between 2004 and April 2021. The search was done according to guidance provided in the Cochrane Handbook for Systematic Reviews of Interventions [21]. Additionally, to search and assess ongoing or unpublished trials, ClinicalTrials.gov and the WHO International Clinical Trials Registry Platform, and the US Food and Drug Administration (FDA) were searched.

The search strategies in PubMed for the MeSH terms and text words was "Child"[Mesh]) AND "Plasmodium falciparum"[Mesh]) OR "Acute malaria" [Supplementary Concept]) OR "Artemether, Lumefantrine Drug Combination/therapeutic use"[Mesh]) OR "Lumefantrine"[Mesh]) OR "dihydroartemisinin" [Supplementary Concept]) OR "piperaquine" [Supplementary Concept]) OR ("Randomized Controlled Trial" [Publication Type] OR "Randomized Controlled Trials as Topic"[Mesh] OR "Controlled Clinical Trial" [Publication Type])) AND ("Drug Therapy"[Mesh] OR "Drug Therapy, Combination"[Mesh] OR "drug therapy" [Subheading])) AND ("Africa"[Mesh] OR "Africa South of the Sahara"[Mesh] OR "Africa, Western"[Mesh] OR "Africa, Southern"[Mesh] OR "Africa, Northern"[Mesh] OR "Africa, Eastern"[Mesh] OR "Africa, Central"[Mesh]. The searching strategies for Cochrane Center for Clinical Trial database (CENTRAL) and Embase are found in Additional file 1.

### Study selection, data collection, and data analysis

**Fig. 1:**
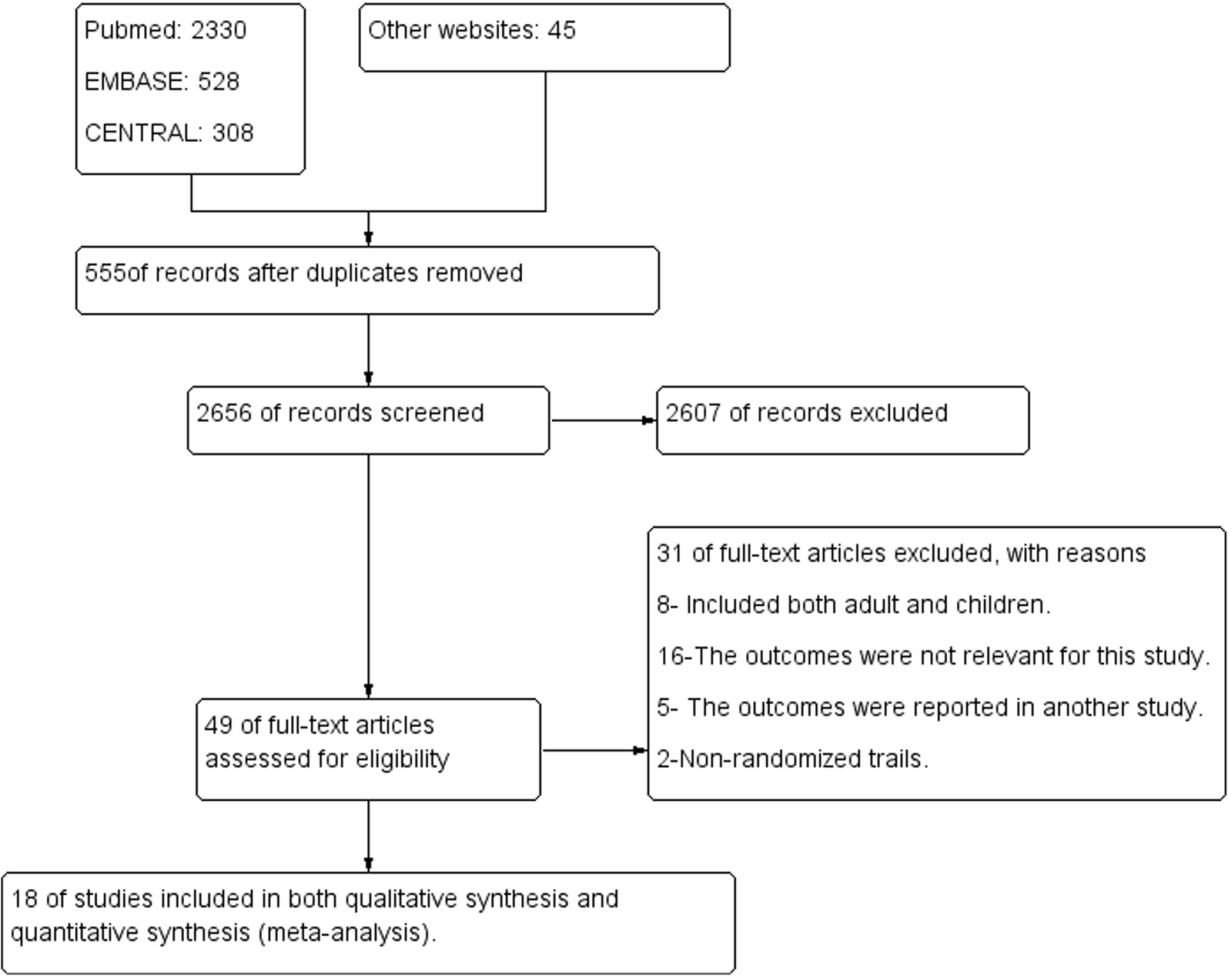
PRISMA study flow diagram of the study

The Cochrane Handbook for Systematic Reviews of Interventions [23] was followed. Furthermore, the software package provided by Cochrane (RevMan 5.4.1) was used. To import the research articles from the electronic databases and remove duplicates, ENDNOTE software version X7 was used. Two authors independently review the results of the literature search and obtained full-text copies of all potentially relevant trials. Disagreements were resolved through discussion. When clarification was necessary, the trial authors were contacted for further information. The screening and selection process was reported in a PRISMA flow chart (Fig. 1).

### Data extraction and management

The title and abstract was produced from the electronic search, and was independently screened by two authors based on RCTs that were assessed human *P. falciparum* malaria. The information collected were trial characteristics including methods, participants, interventions, and outcomes as well as data on dose and drug ratios of the combinations. Also, relevant information such as title, journal, year of publication, publication status, study design, study setting, malaria transmission intensity, follow-up period, sample size, funding of the trial or sources of support, baseline characteristics of study subjects and adverse events including serious AEs were extracted from each article using the well-prepared extraction format in the form of a table adapted from Cochrane and modified to make suitable for this study.

Furthermore, the number of participants randomized, and the number analysed in each treatment group for each outcome were also collected. One author independently extracted data and information collected was cross-checked by another investigator. The number of participants experiencing the event and the number of participants in each treatment group were documented.

### Assessment of risk of bias in included studies

The risk of bias for each trial was evaluated by two review authors independently using the Cochrane Collaboration's tool for assessing the 'Risk of bias' [21]. To decrease the risk of bias amongst six domains: sequence generation; allocation concealment; blinding (of participants, personnel, and outcome assessors); incomplete outcome data; selective outcome reporting; and other sources of bias, this guidance were used. The risks were classified as high risk, unclear risk, and low risk.

### Measures of treatment effect

The main outcomes in this review were total of patients who experienced one or more adverse events. A number of patients with AEs from the studies were combined and presented using risk rations accompanied by 95% CIs.

### Assessment of heterogeneity

Heterogeneity among the included trials was assessed by inspecting the forest plots and the Cochrane Q and I^2^ statistic used to measure heterogeneity among the trials in each analysis, the Chi^2^ test with a P < 0.10 to indicate statistical significance was used, and the results were interpreted following Cochrane Handbook for Systematic Reviews of Interventions Version 6.0, Chapter 10: Analyzing data and undertaking meta-analyses [24].

### Assessment of reporting bias

To assess the possibility of publication bias, funnel plots for asymmetry (Egger’s test P < 0.05) were used [25].

### Data synthesis

The meta-analyses was done consistent with the recommendations of Cochrane [23]. To aid interpretation, identity codes were given to included trials together with the first author, year of publication, and three first letter of the country where the trial being conducted. Trials were shown in forest plots in chronological order of the year the trials were published. A random-effects model was used, as trials were done by different researchers, operating independently, and it could be implausible that all the trials had functionally equivalence, with a common effect estimate.

### Sensitivity analysis

To investigate the strength of the methodology used in the primary analysis and to restore the integrity of the randomization process, a series of sensitivity analyses were conducted using following steps were used: adding and excluding trials which were classified as high risk for bias back into the analysis in a stepwise fashion, and to assess the influence of small-study effects on the results of our meta-analysis, fixed-effect and random-effects estimates of the intervention effect were compared.

### Quality of evidence

Quality of evidence was assessed using GRADE criteria and the GRADE pro software [26]. The results were presented in a ‘Summary of Findings’ table. Randomized trials are initially categorized as high quality but downgraded after assessment of five criteria [27]. The levels of evidence were defined as 'high', 'moderate', 'low', or 'very low'. The recommendations of Section 8.5 and Chapter 13 of the Cochrane Handbook for Systematic Reviews of Interventions was followed [28]. The imprecision was judged based on the optimal information size criteria and CI [29].

## Results

A total of 3211 studies through the databases were searched, of which 49 full-text trials for eligibility were assessed and 18 of them fulfilled the inclusion criteria for meta-analysis and for qualitative analysis (see Fig. 1).

### Characteristics of included studies

In this review, 18 studies were included, which enrolled 10,498 participants with uncomplicated *P. falciparum* malaria were included Table 1.

**Table 1:** Characteristics of included studies

S. No	Study ID		Study design			Study setting and period				Transmission		Follow up		Subjects							Patient important outcome	DHA-PQ	AL			
														Number of participants					Inclusion age							
														DHA-PQ			AL									
1	Kamya-2007-UGA [30]		Single-blind, RCT			Rural health center, March,2006-July, 2006				High transmission		42 days		253			256		6 months–10 years		Vomiting	65	65			
																					Diarrhoea	25	19			
																					Anorexia	90	91			
																					Abdominal pain	19	20			
																					weakness/malaise	85	103			
																					Cough	136	133			
																					Coryza	127	121			
																					Pruritus	14	22			
																					SAE	4	2			
2	Zongo-2007-BNF [31]		Single blind RCT			Government health dispensaries, August 2006-January 2007			High transmission			42 days		196			197		6 months–10 years		Early vomiting	7	3			
																					Vomiting	20	27			
																					Diarrhoea	14	13			
																					Anorexia	8	6			
																					Abdominal pain	10	21			
																					Cough	49	52			
																					Weakness/Malaise	5	3			
																					Pruritus	5	11			
																					Headache	11	22			
3	Mens-2008-KEN [32]		Open label RCT			Health center, Apr 2007 to Jul 2007			High transmission			28 days		73			73		6 months–12 years		Headache	43	39			
																					Abdominal pain	25	26			
																					Weakness	19	30			
																					Anorexia	8	10			
																					Diarrhoea	9	7			
																					Cough	16	17			
																					Vomiting	11	9			
																					Pruritus	4	3			
																					SAE	1	0			
4	Yeka-2008-UGA [33]		Single-blind, RCT			Health center, August 2006-April 2007			N/A			42 days		234			227		6 months–10 years		Vomiting	35	35			
																					Diarrhoea	26	23			
																					Anorexia	47	49			
																					Abdominal pain	17	24			
																					Weakness/malaise	28	27			
																					Cough	164	150			
																					Coryza	159	150			
																					Pruritus	8	3			
																					SAE	5	2			
5	Bassat-2009-AFR [34]		Open-label, RCT			Four rural sites and one peri-urban site, August 2005 and July 2006.						Mesoendemic		1038			510		6–59 months		Early vomiting	22	4			
																					Vomiting	71	35			
																					Splenomegaly	41	19			
																					Hepatomegaly	6	3			
																					5 Prolonged QTc interval (Fridericia’s correction)	2	1			
																					Electrocardiogram QT prolonged	26	13			
																					Urticarial	1	2			
																					Hypersensitivity	2	1			
																					Neutropenia	18	12			
																					Alanine aminotransferase increased	20	19			
																					Electrocardiogram QT prolonged	26	13			
																					SAE	18	5			
6	Arinaitwe-2009-UGA [35]		Open-label RCT			Local antenatal clinics in Tororo, August 2007-July 2008			High transmission			63 days		119			111		6 weeks–12 months		Vomiting	23	20			
																					Diarrhoea	79	86			
																					Anorexia	3	0			
																					Weakness	1	0			
																					Cough	177	153			
																					Pruritus	0	0			
																					SAE	3	1			
7	Borrmann-2011–KEN [36]		Not described, RCT			Pingilikani study site, September 2005 to April 2008			Perennial transmission			84 days		233			241		6–59 months		Early vomiting	7	4			
8	Nambozi-2011-ZAM [37]		Open-label, RCT			Peri-urban health centers, September 2005 and May 2006			Mesoendemic			42 days		203			101		6–59 months		Anorexia	14	8			
																					Cough	42	15			
																					Diarrhoea	14	4			
																					Fever	24	14			
																					Respiratory tract Infection	22	9			
																					Vomiting	5	4			
																					SAE	4	3			
9	4ABC-2011-AFR [38]		Open label, RCT			Rural, urban or health facilities, 9 July 2007 and 19 June 2009			Mesoendemic, perennial and high transmission			63 days		1475			1226		6–59 months		Death up to day 63	1	3			
																					Hepatomegaly	5	8			
																					Splenomegaly	88	80			
																					Anemia	141	38			
																					Diarrhoea	166	142			
																					Vomiting	123	102			
																					Pyrexia	371	339			
																					Hgb decrease	103	83			
																					Anorexia	130	121			
																					Cough	470	387			
																					ALAT above normal range at day 0	10	16			
																					ALAT above normal range at day 7	3	4			
																					ALAT above normal range at day 28	4	1			
																					Creatinine above normal range at day 0	2	0			
																					Creatinine above normal range at day 7	0	0			
																					Creatinine above normal range at day 28	0	2			
																					SAE	10	6			
10	Agarwal -2013-KEN [39]		An open label RCT			District hospital, October 2010 to August 2011			High transmission			42 days		137			137		6–59 months		Early vomiting	7	5			
																					SAE	1	2			
11	Ogutu-2014-KEN [40]		Open-label, RCT			Nyando District hospital, March, 2010-30 November, 2011			Not described			42 days		227			227		6–59 months		Cough	40	37			
																					Anemia	8	10			
																					Fever	14	7			
																					Tinea capitis	12	10			
																					Rhinitis	13	4			
																					Gastroenteritis	5	9			
																					Loss of appetite	6	3			
																					Otitis media	5	7			
12	Onyamboko-2014-DRC [41]		Open label, RCT			Urban district of Kinshasa (DRC) (Hospitals), September 2011 and November 2012			Intense and perennial			42 days		228			228		3–59 months		Early vomiting	21	5			
																					Vomiting	17	2			
173	Kakuru-2014-UGA [42]		Not described, RCT			District Hospital, August 2007 and April 2008			High transmission			28 days		21			22		6 weeks -12 months		Vomiting	8	18			
																					Diarrhoea	27	23			
																					Anorexia	6	4			
																					Weakness/malaise	2	2			
																					Cough	64	74			
14	Nji-2015-CAM [43]		Open-label, RCT			Two distinct ecological regions, 2009 to April 2013			Low to moderate transmission			42 days		288			144		6 months-10 years		Abdominal pain	13	5			
																					Anorexia	12	1			
																					Diarrhoea	9	4			
																					Vomiting	27	8			
																					Fatigue	4	3			
																					Fever	3	2			
																					Cough	18	9			
																					Joint pain	2	2			
																					Rash	16	4			
																					SAE	0	1			
15	Ursing-2016-GUB [44]		Open-label, RCT			Bandimand Belem Health Centers, November 2012 and July 2015			Low to high transmission			42 days		157			155		6 months–15 years		Early vomiting	7	4			
16	Grandesso-2018-NIG [45]		Open label, RCT			Health center, 7 June 2013 and 22 September 2014			Not reported			42 days		221			221		6–59 months		Early vomiting	1	0			
																					Fever	94	94			
																					Cough	36	22			
																					Rhinorrhea	27	17			
																					Diarrhoea	14	15			
																					Conjunctivitis	7	15			
																					Pyoderma	6	6			
																					Vomiting	6	5			
																					Anorexia	4	1			
																					Abdominal pain	0	1			
																					Hepatomegaly	1	0			
																					Splenomegaly	2	1			
																					Another AE	40	45			
																					SAE	2	1			
																					Early vomiting	1	2			
17	Yeka-2019-UGA [46]			Single-blind RCT		Health center and Hospital, October 2015-December, 2016	High transmission				42 days			299		300			6–59 months		Vomiting	56	61			
																					Diarrhoea	155	114			
																					Anorexia	12	3			
																					Abdominal pain	41	45	Cough	233	203
																					Headaches	18	24	Pallor	22	13
																					weakness/malaise	42	33	Skin rash	56	42
																					Cough	233	203	Pruritus	24	16
																					Pallor	22	13	SAE	6	6
																					Skin rash	56	42			
																					Pruritus	24	16			
																					SAE	6	6			
18		Gansane-2021-BNF [47]			Open label, RCT	Primary health facility and district hospital, November 2017 to September 2018		Moderate to high transmission					42 days		360			360		6–59 months	Itchiness	0	1			
																					Otitis media	0	1			
																					Cough	17	21			
																					Abdominal pain	13	4	Vomiting	33	54
																					Skin rash	3	2	SAE	0	1
																					Furunculosis	1	0			
																					Vomiting	33	54			
																					SAE	0	1			

### Characteristics of excluded studies

Thirty one studies were excluded with reason, Additional file 2.

### Methodological quality and risk of bias

**Fig. 2:**
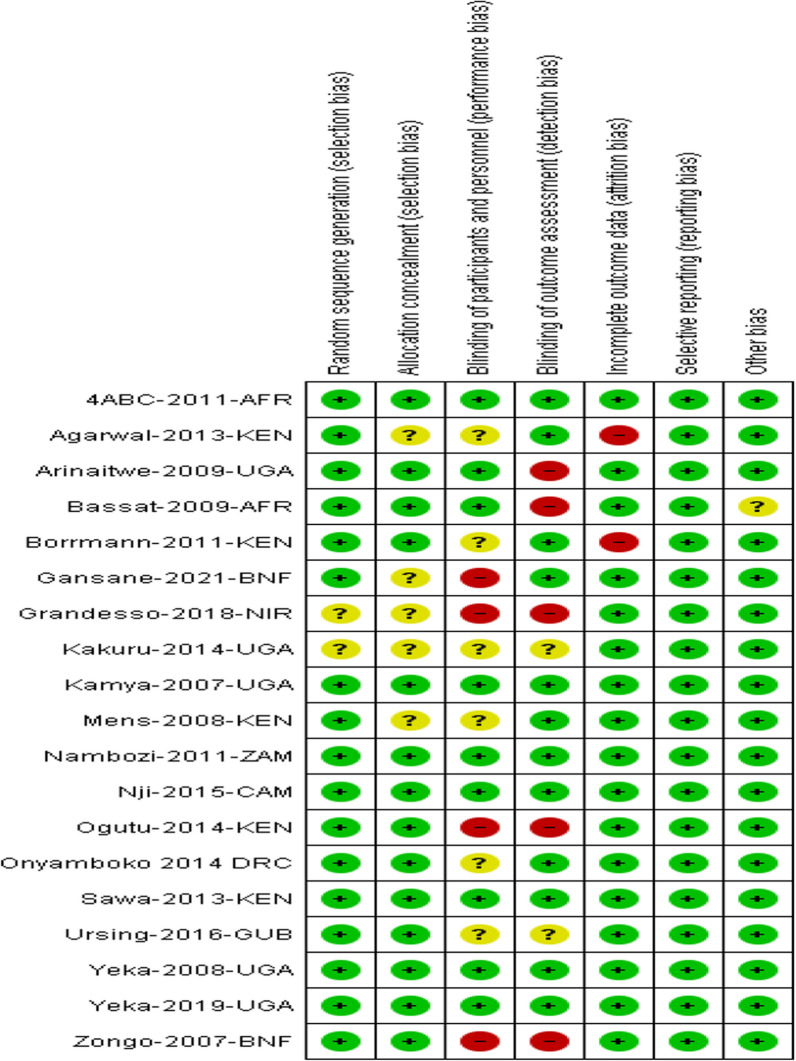
A summary of review authors' judgments about each risk of bias item for each included study

The 'Risk of bias' assessments were summarized in Fig. 2.

### Adverse events

#### Gastrointestinal adverse events

##### Early vomiting

**Fig. 3:**
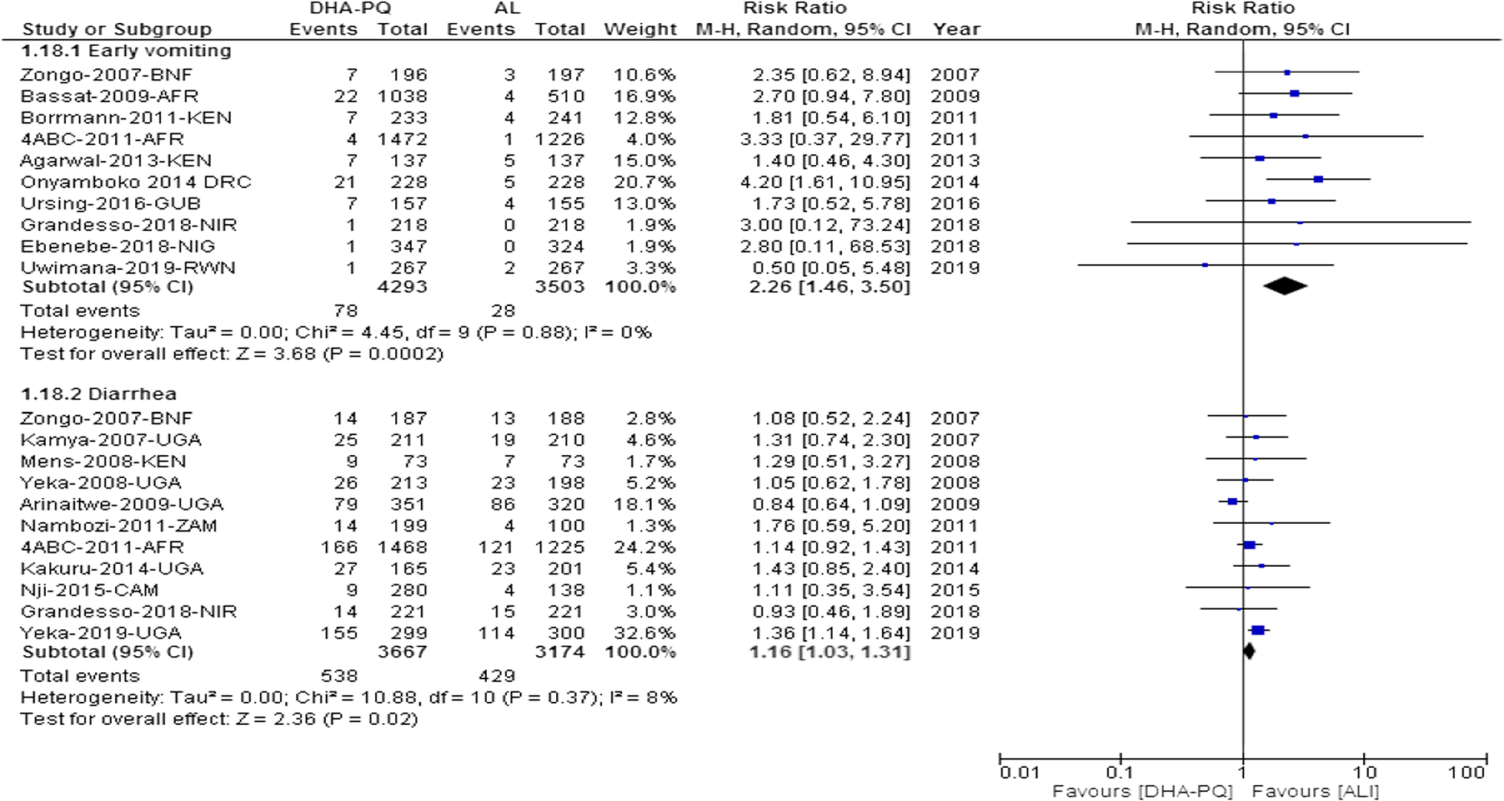
Forest plot of comparison with dihydroartemisinin-piperaquine and artemether-lumefantrine for treatment of uncomplicated *plasmodium falciparum* malaria among children in Africa on gastrointestinal adverse events

The relative risk of early vomiting in patients treated with the DHA-PQ was higher than AL (RR 2.26, 95% CI 1.46 to 3.50; participants = 7796; studies = 10; I^2^ = 0%, *high quality of evidence,* Fig. 3).

##### Publication bias

The funnel plot showed that all studies lay symmetrically around the pooled effect estimate implying that there was no publication bias (P = 0.5, Additional file 3).

##### Diarrhoea

Similarly, the relative risk of early vomiting in patients treated with the DHA-PQ was higher than AL (RR 1.16, 95% CI 1.03 to 1.31; participants = 6841; studies = 11; I^2^ = 8%, *high quality of evidence,* Fig. 3).

##### Publication bias

The funnel plot showed that all studies lay symmetrically around the pooled effect estimate implying that there was no publication bias (P = 0.9, Additional file 4).

#### Other gastrointestinal adverse events

**Fig. 4:**
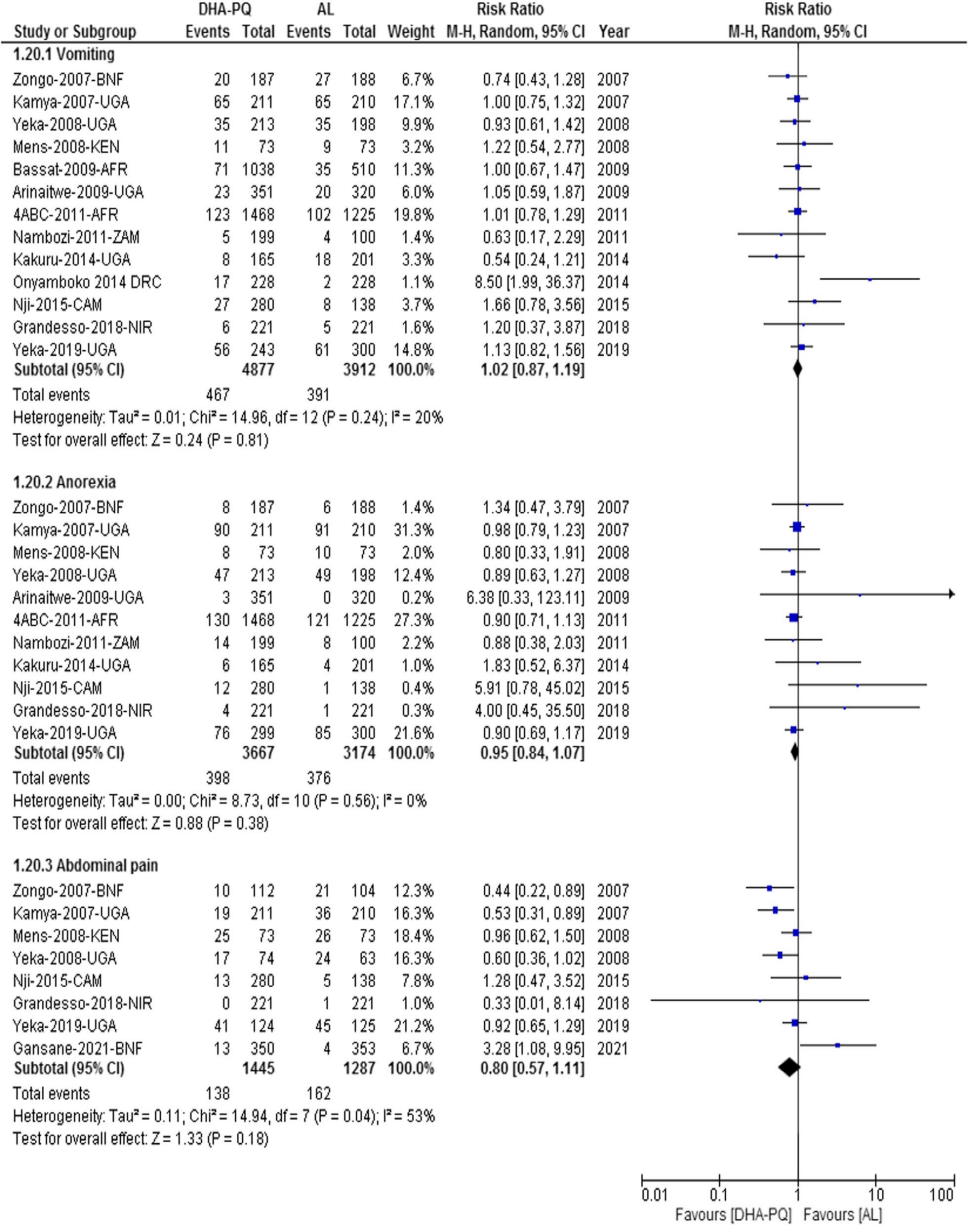
Forest plot of comparison: dihydroartemisinin-piperaquine versus artemether-lumefantrine for treatment of uncomplicated *Plasmodium falciparum* malaria among children in Africa, outcome: Gastrointestinal adverse events

The risk of vomiting did not have significant difference between the two treatment groups (RR 1.02, 95% CI 0.87 to 1.19; participants = 8789; studies = 13; I^2^ = 20%, *high quality of evidence*, Fig. 4). Similarly, there was no significant difference between the two treatment groups on the relative risk of anorexia (RR 0.95, 95% CI 0.84 to 1.07; participants = 6841; studies = 11; I^2^ = 0%, *high quality of evidence)*, abdominal pain (RR 0.80, 95% CI 0.57 to 1.11; participants = 2732; studies = 8; I^2^ = 53%, *high quality of evidence,* Fig. 4), gastroenteritis (RR 0.57, 95% CI 0.19 to 1.68; participants = 469, and loss of appetite (RR 2.06, 95% CI 0.52 to 8.14; participants = 469; studies = 1, [40]).

#### Cardio-respiratory adverse events

##### Cough

**Fig. 5:**
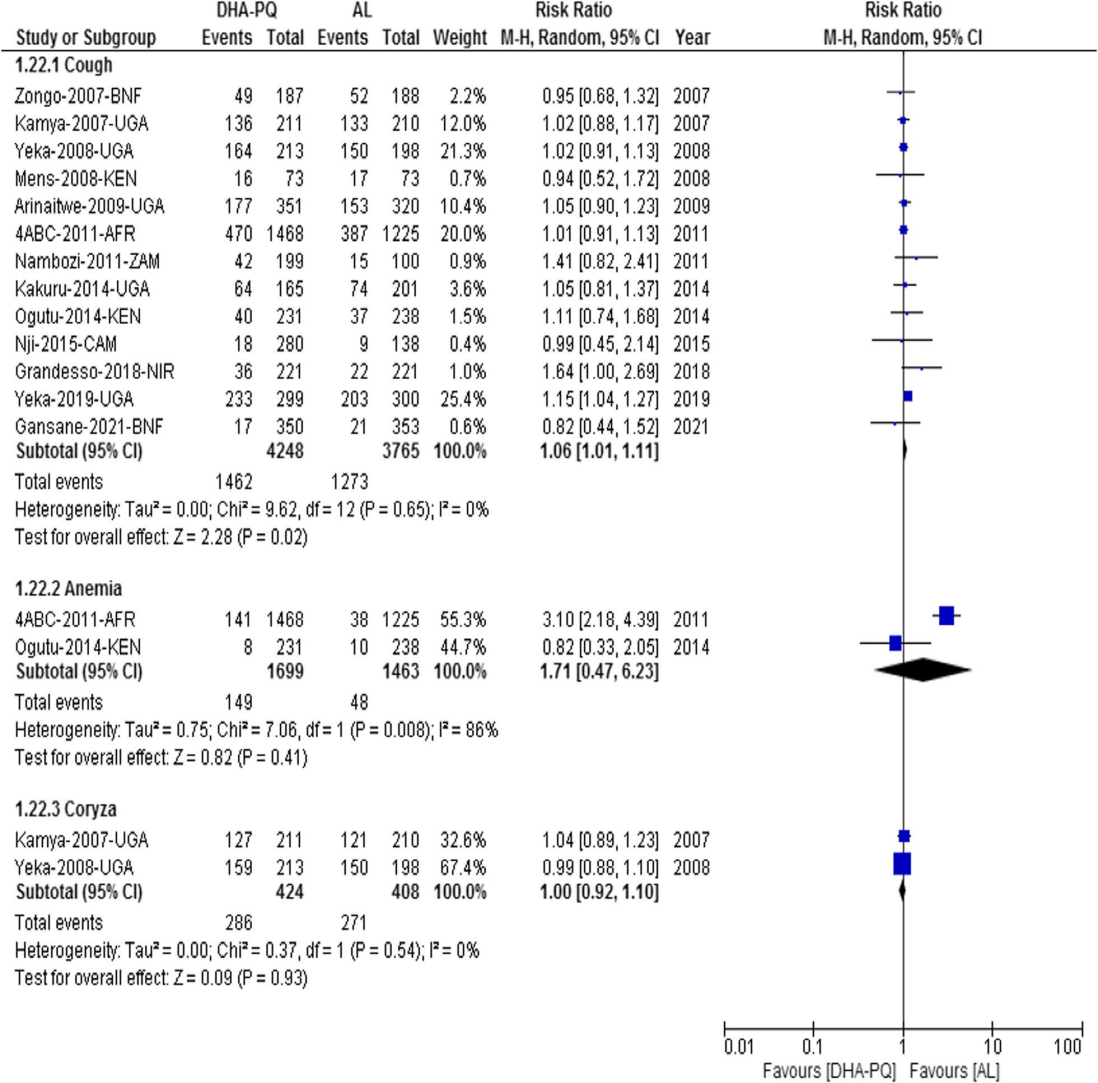
Forest plot of comparison between dihydroartemisinin-piperaquine and artemether-lumefantrine for treatment of uncomplicated *Plasmodium falciparum* malaria among children in Africa on cardio-respiratory adverse events

Cough was the most common cardio-respiratory adverse event, and significantly higher number of participants from DHA-PQ treatment group experienced cough (RR 1.06, 95% CI 1.01 to 1.11; participants = 8013; studies = 13; I^2^ = 0%, *high quality of evidence,* Fig. 5).

##### Publication bias

The funnel plot shows that all studies lie symmetrically around the pooled effect estimate implying that there was no publication bias (P = 0.84, Additional file 5).

#### Other cardiorespiratory and hematological adverse events

The relative risk of developing coryza did not have significant difference between the two treatment groups (RR 1.00, 95% CI 0.92 to 1.10; participants = 832; studies = 2; I^2^ = 0%, Fig. 5). In addition, the relative risk of respiratory adverse events such as rhinorrhea, respiratory tract infection, rhinitis, and pallor was not significantly different between the two treatment groups (RR 1.59, 95% CI 0.89 to 2.83; participants = 442; studies = 1, [45]), (RR 1.23, 95% CI 0.59 to 2.57; participants = 299; studies = 1, [37]), (RR 3.35, 95% CI 1.11 to 10.12; participants = 469; studies = 1, [40]), 95% CI 0.91 to 1.92; participants = 1548; studies = 1, [34]). Similarly, the relative risk of cardiac adverse events like QTc interval prolongation (Fridericia’s correction and Bazett’s correction) was not significantly different between the two treatment groups (RR 0.98, 95% CI 0.51 to 1.90; participants = 1548; studies = 1, [34] and (RR 0.98, 95% CI 0.09 to 10.81 and RR 1.32, 95% CI 0.91 to 1.92, participants = 1548, studies = 1, [34]).

#### Neuropsychiatry adverse event

##### Weakness/malaise

**Fig. 6:**
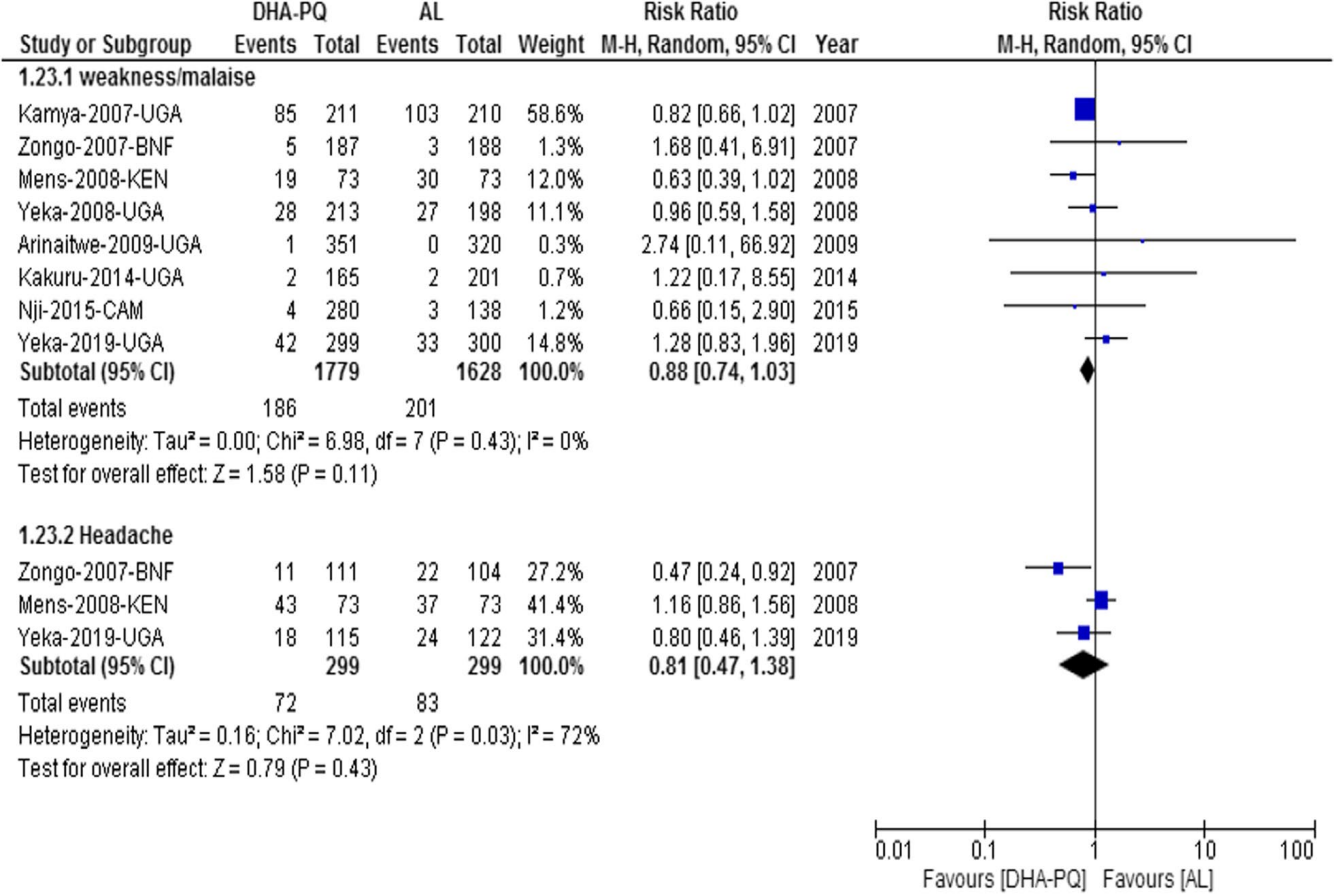
Forest plot of comparison: dihydroartemisinin-piperaquine versus artemether-lumefantrine for treatment of uncomplicated *Plasmodium falciparum* malaria among children in Africa, outcome: Neuropsychiatry adverse event

The relative risk of developing weakness or malaise was not significantly different between the two treatment groups (RR 0.88, 95% CI 0.74 to 1.03; participants = 3407; studies = 8; I^2^ = 0%, *high quality of evidence*, Fig. 6). Also, the relative risk of headache was not significantly different between the two treatment groups (RR 0.81, 95% CI 0.47 to 1.38; participants = 598; studies = 3; I^2^ = 72%, Fig. 6).

#### Musculoskeletal/dermatological adverse events

**Fig. 7:**
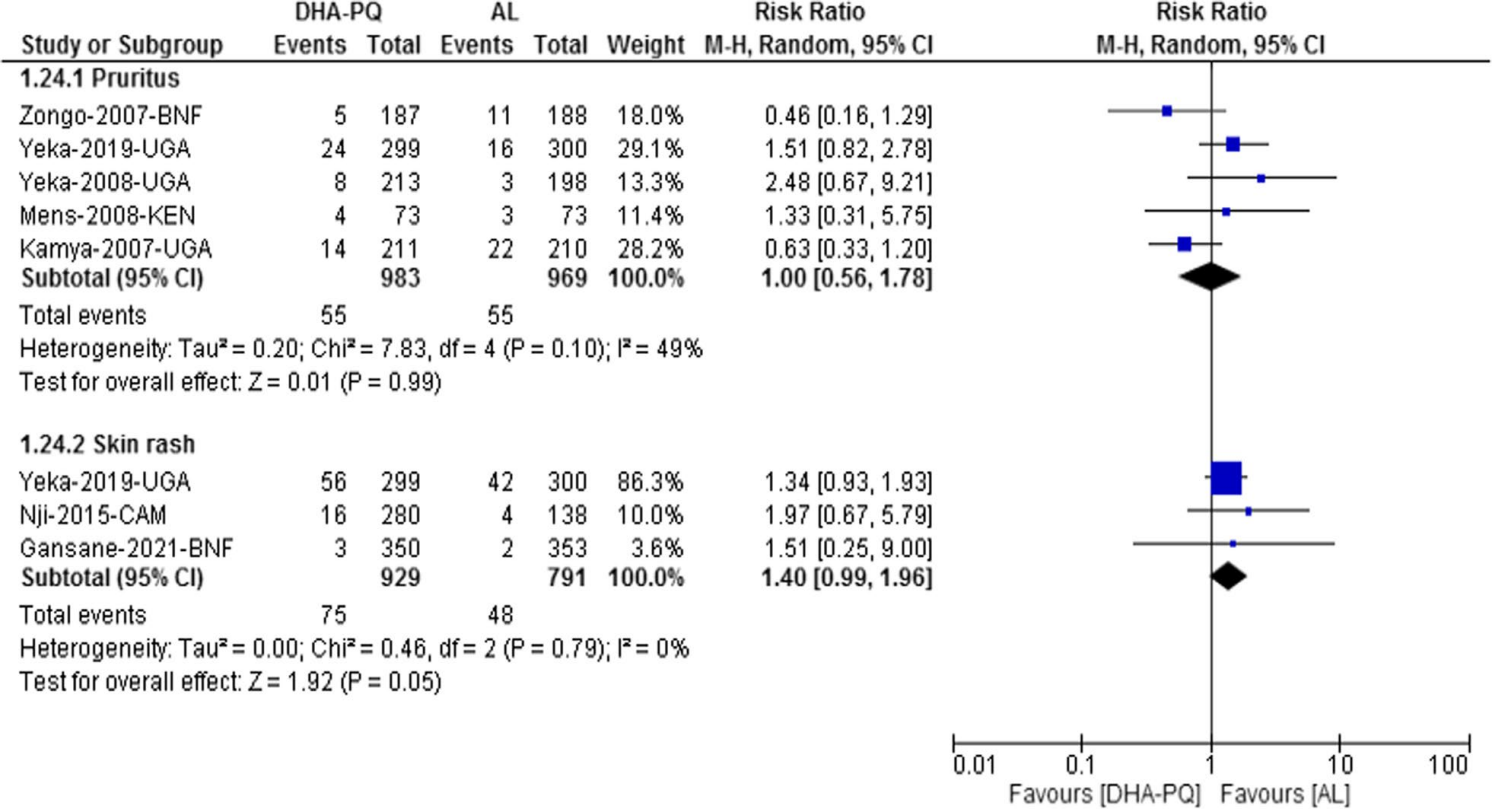
Forest plot of comparison: dihydroartemisinin-piperaquine versus artemether-lumefantrine for treatment of uncomplicated *Plasmodium falciparum* malaria among children in Africa, outcome: Musculoskeletal/dermatological adverse events

Pruritus was the most common dermatological adverse event, and the relative risk of developing pruritus was not significantly different between the two treatment groups (RR 1.00, 95% CI 0.56 to 1.78; participants = 1952; studies = 5; I^2^ = 49%, *moderate quality of evidence*, Fig. 7). Also, the relative risk of developing skin rash was not significantly different between the two treatment groups (RR 1.40, 95% CI 0.99 to 1.96; participants = 1720; studies = 3; I^2^ = 0%, Fig. 7).

#### Other musculoskeletal/dermatological adverse events

The relative risk of musculoskeletal or dermatological adverse events such as: skin and subcutaneous disorder, urticarial, hypersensitivity, pyoderma, conjunctivitis, joint pain, tinea-capitis, itchiness, frunculosis was not significantly different between the two treatment groups (RR 1.19, 95% CI 0.78 to 1.80; participants = 1548; studies = 1, [34]), (RR 0.25, 95% CI 0.02 to 2.70; participants = 1548; studies = 1, [34]), (RR 0.98, 95% CI 0.09 to 10.81; participants = 1548; studies = 1, [33]), (RR 1.00, 95% CI 0.33 to 3.05; participants = 442; studies = 1, [45]), (RR 0.47, 95% CI 0.19 to 1.12; participants = 442; studies = 1, [45]), (RR 0.49, 95% CI 0.07 to 3.46; participants = 418; studies = 1, [43]), (RR 1.24, 95% CI 0.54 to 2.81; participants = 469; studies = 1, [40]), (RR 0.34, 95% CI 0.01 to 8.22; participants = 703; studies = 1 [47],) and (RR 3.03, 95% CI 0.12 to 74.02; participants = 703; studies = 1, [47]), respectively.

#### Other adverse events

##### Pyrexia

**Fig. 8:**
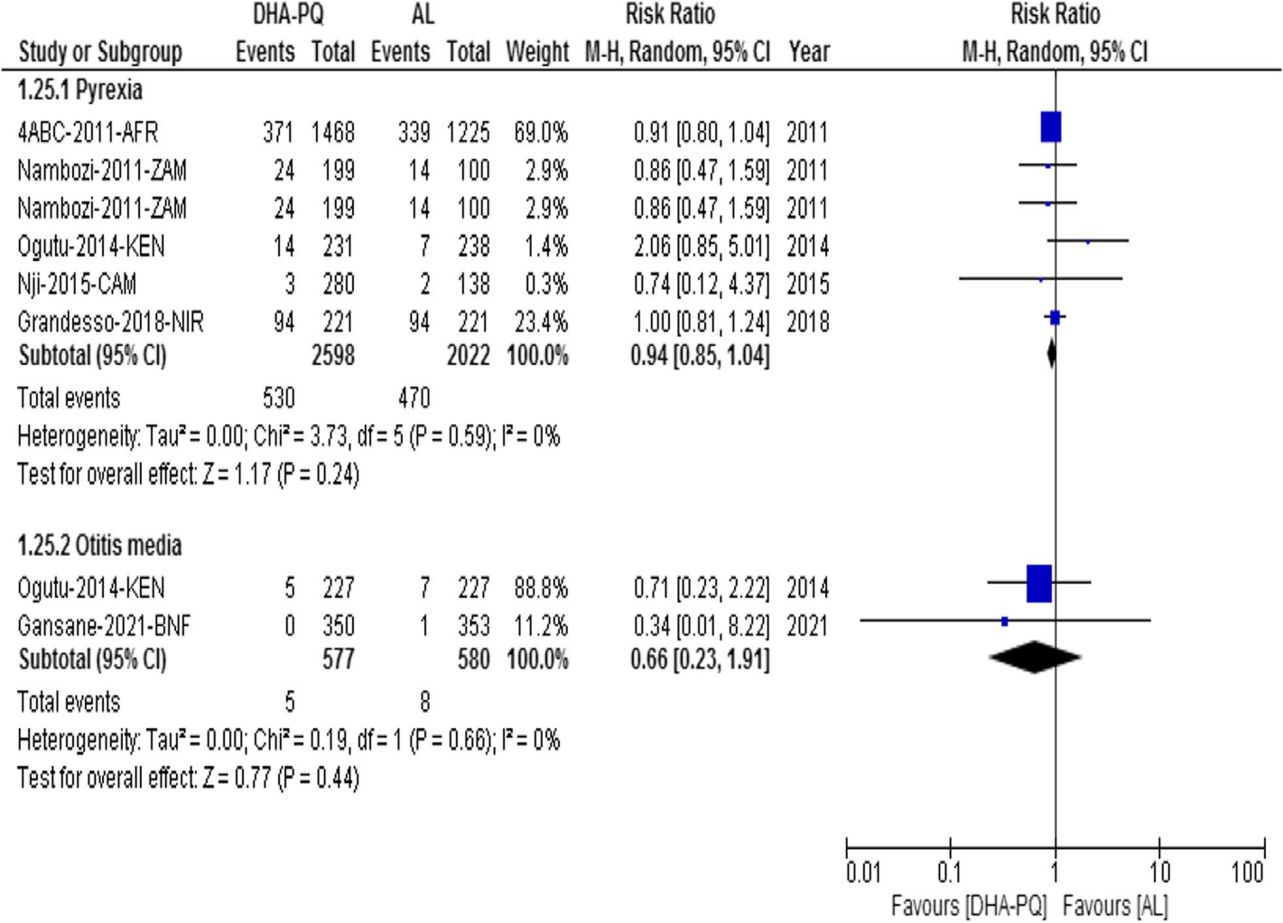
Forest plot of comparison: dihydroartemisinin-piperaquine versus artemether-lumefantrine for treatment of uncomplicated *Plasmodium falciparum* malaria among children in Africa, outcome: Other Adverse events

The relative risk of pyrexia was the same in both treatment groups (RR 0.94, 95% CI 0.85 to 1.04; participants = 4620; studies = 6; I^2^ = 0%, Fig. 8). Similarly, the relative risk of otitis media was the same in both treatment groups (RR 0.66, 95% CI 0.23 to 1.91; participants = 1157; studies = 2; I^2^ = 0%, Fig. 8).

##### Serious adverse event

**Fig. 9:**
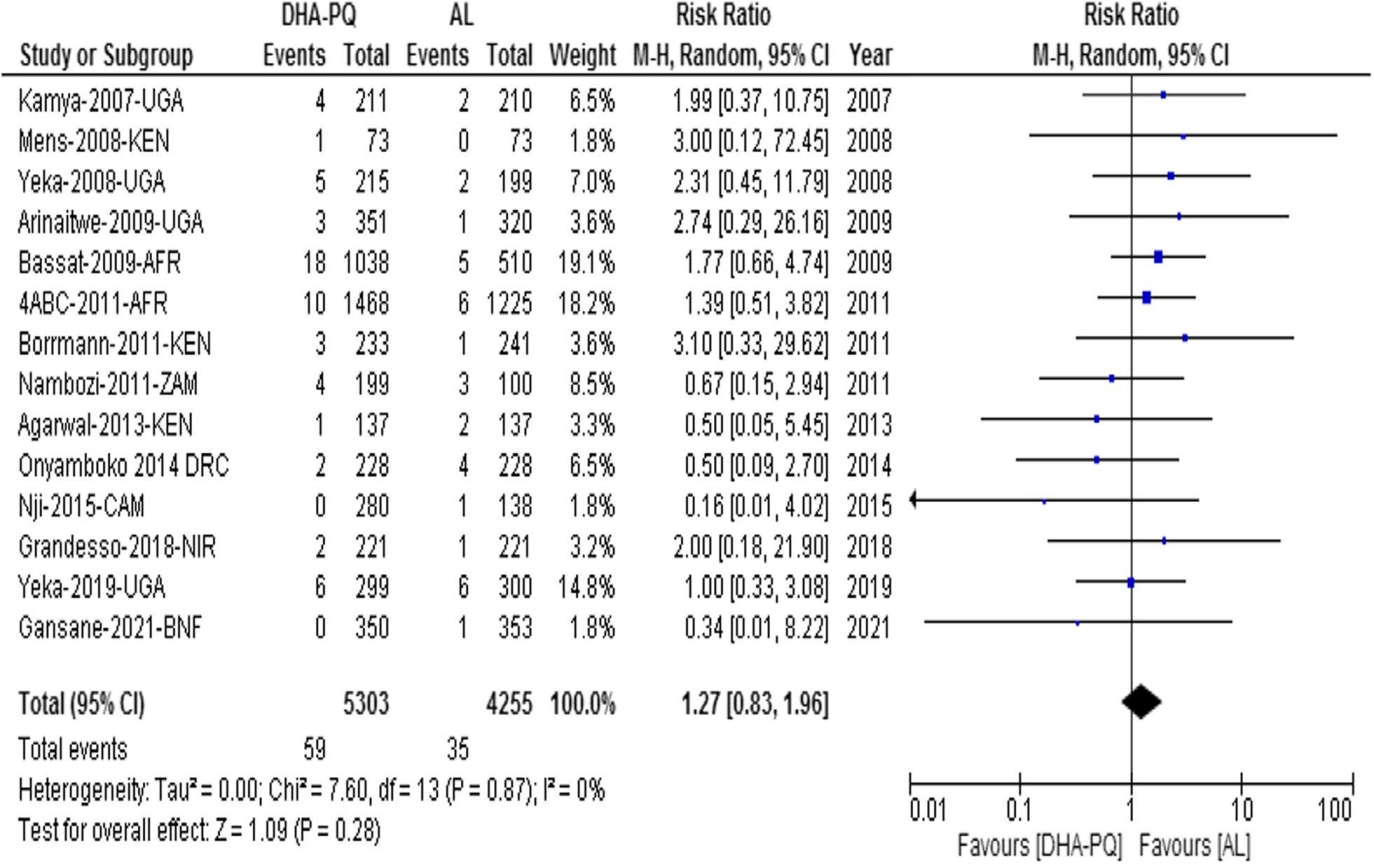
Forest plot of comparison between dihydroartemisinin-piperaquine and artemether-lumefantrine for treatment of uncomplicated *Plasmodium falciparum* malaria among children in Africa on serious adverse event (including death)

Fourteen studies reported 59 serious adverse events in the DHA-PQ and 35 in the AL treatment groups. However, the distributions of serious adverse events were not significantly different in the two treatment groups (RR 1.27, 95% CI 0.83 to 1.96; participants = 9558; studies = 14; I^2^ = 0%, *high quality of evidence*, Fig. 9). Eight deaths were reported from two multi-center trials, and the cause of death for seven of them was sepsis, severe malaria, and severe diarrhoea. But, the causal relationship of the study drug and death of one participant didn’t rule out. All serious adverse events were likely a consequence of malaria and judged to be unrelated to study medications.

##### Publication bias

The funnel plot showed that all studies lay symmetrically around the pooled effect estimate implying that there was no publication bias (P = 0.50, Additional file 6).

### Quality of the evidence

The quality of the evidence in this review assessed using the GRADE approach and presented the evidence in three summary of findings tables for safety (Summary of findings for the main comparison; Additional file 7). The quality of evidence on comparative adverse effects and serious adverse events; early vomiting, diarrhoea, and cough were slightly more frequent in the DHA-PQ arm (*high quality of evidence)*. Generally, the quality of evidence of safety of the two treatments was high quality.

## Discussion

In this study both drugs were well tolerated by children. There were comparable occurrences of adverse events in both treatment arms. But, early vomiting, diarrhoea, and cough were common were significantly more frequent in patients who were treated with the DHA-PQ than that of AL (*high quality of evidence*). All serious adverse events were not related to study medications. Eight deaths have occurred in all studies. But, all serious adverse events were consistent with malaria symptoms and judged to be unrelated to study medication.

As also seen in one study from Papua New Guinea, the overall frequency of adverse events were slightly higher in DHA-PQ treatment arm than that of AL [48]. However, cough was more frequent in patients who were treated with AL, but headache and runny nose were common in DHA-PQ treatment group [48]. A recent review on the efficacy and safety of the two ACT’s also reported that cough, anorexia, diarrhoea, and vomiting were the most common adverse events. In this review more patients from DHA-PQ treatment arm had cough than that of AL [49] and similarly, gastrointestinal adverse events were more frequent in patients who were treated with DHA-PQ in another study done in South East Asia and Africa [50–53]. Studies from the Thailand-Myanmar border [54, 55] and elsewhere in Africa [56–58] have reported that DHA-PQ cause drug induced electrocardiographic QT prolongation, but a recent study also reported that the QT prolongation caused by piperaquine is not associated with an increased risk of sudden death [59]. In breastfeeding infants DHA–PQ has previously been linked to an increased risk of vomiting [60]. The mechanism accountable for the increased risk of early vomiting among breastfeeding participants treated with DHA–PQ is not known.

However, the temporal relationship suggests that the susceptibility of gastric mucosa of breastfed infants could be related to the pro-emetic effect of piperaquine than that in weaned infants [60]. To determine whether the co-administered milk may also affect this interaction further assessment might be needed [60]. However, the absence of effect with AL implies that the mechanism is given to DHA–PQ, most likely piperaquine [17]. Regardless of the treatment groups, most of these adverse events are associated with age (≤ 18 years), efavirenz-based ART [52], efavirenz-based ART [53], and administration of DHA-PQ with food could increase piperaquine exposure and it needs to be administered in fasting state [53, 54, 61].

Most of the RCTs reported AEs rather than adverse reactions of the antimalarial drugs. This made it difficult to determine the causal relationship between the antimalarial drugs and the AEs. It was, therefore, difficult to determine whether an adverse event is symptomatic of the disease or drug related. In some other studies, safety reporting was either selective or inadequate, with some authors failing to indicate the severity of AEs. Some of these limitations have been identified in studies evaluating the quality of safety reporting in RCTs.

## Conclusion

From this review, it can be concluded that early vomiting, diarrhoea, and cough were common were significantly more frequent in patients who were treated with the DHA-PQ than that of AL, and both drugs are well tolerated. More studies comparing AL with DHA-PQ are needed to determine the comparative safety of these drugs.

## Supplementary Information

**Additional file 1.** Detailed search strategy.

**Additional file 2.** Characteristics of excluded studies.

**Additional file 3.** Funnel plot of comparison: dihydroartemisinin-piperaquine versus artemether-lumefantrine for treatment of uncomplicated *Plasmodium falciparum* malaria among African children, outcome: Gastrointestinal adverse events (early vomiting).

**Additional file 4.** Funnel plot of comparison: dihydroartemisinin-piperaquine versus artemether-lumefantrine for treatment of uncomplicated *Plasmodium falciparum* malaria among African children, outcome: Gastrointestinal adverse events (diarrhoea).

**Additional file 5.** Funnel plot of comparison: dihydroartemisinin-piperaquine versus artemether-lumefantrine for treatment of uncomplicated *Plasmodium falciparum* malaria among African children, outcome: Cough.

**Additional file 6**. Funnel plot of comparison: dihydroartemisinin-piperaquine versus artemether-lumefantrine for treatment of uncomplicated *Plasmodium falciparum* malaria among African children, outcome: Serious adverse event (including death).

**Additional file 7.** GRADE summary of findings table on adverse events and serious adverse events.

## Data Availability

All relevant data are within the manuscript and its supporting information files.

## Abbreviations

AE: Adverse event
ACT: Artemisinin-based combination therapy
AL: Artemether-lumefantrine
ART: Antiretroviral therapy
BW: Body weight
CENTRAL: Cochrane Central Register of Controlled Trials
CI: Confidence interval
DHA-PQ: Dihydroartemisinin-piperaquine
GADE: Grading of recommendations assessment development and evaluations
PICO: Population intervention comparison and outcome
PRISMA: Preferred reporting items for systematic reviews and meta-analyses
RCTs: Randomized control trials
RR: Risk ratio
WHO: World Health Organization
