# Supplementary material for: Safety of dihydroartemisinin-piperaquine versus artemether-lumefantrine for the treatment of uncomplicated Plasmodium falciparum malaria among children in Africa: a systematic review and meta-analysis of randomized control trials
Source: Malar J. 2022 Jan 4;21:4. doi: 10.1186/s12936-021-04032-2 (PMC8725395; doi:10.1186/s12936-021-04032-2)
Supplement: Supplementary file 2 — Additional file 2. Characteristics of excluded studies. [file 12936_2021_4032_MOESM2_ESM.docx]

Additional file S 2: Characteristics of excluded studies

| **No** | **Studies** | **Reason for exclusion** |
| --- | --- | --- |
|  | Adam-2010-SUD [1] | Both children and adults were enrolled in this trial. Children’s outcome didn’t report. |
|  | Agarwal -2013-KEN [35] | The outcomes were not relevant for this study. |
|  | Conrad-2014-UGA [3] | The outcomes were not relevant for this study. |
|  | Creek-2010-UGA[4] | The outcomes were reported in another study. |
|  | Dama-2018-MAL [5] | Both children and adults were enrolled in this trial. Children’s outcome didn’t report. |
|  | Davlantes-2018-ANG [6] | Both children and adults were enrolled in this trial. Children’s outcome didn’t report. |
|  | Diallo-2020-SEN [7] | Both children and adults were enrolled in this trial. Children’s outcome didn’t report. |
|  | Funck -2019 [9] | The outcomes were not relevant for this study. |
|  | Green-2016 [10] | The outcomes were not relevant for this study. |
|  | Ishengoma-2019-TAN [11] | The outcomes were not relevant for this study. |
|  | Kakolwa-2018-TAN [12] | The outcomes were not relevant for this study. |
|  | Kakuru-2013-UGA [13] | The outcomes were reported in another study. |
|  | Katrak-2009-UGA [14] | The outcomes were reported in another study. |
|  | Meremikwu-2013-NIG [15] | The outcomes were not relevant for this study. |
|  | Menan-2011- AFR [3] | Both children and adults were enrolled in this trial. Children’s outcome didn’t report. |
|  | Muhindo-2014-UGA [16] | The outcomes were not relevant for this study. |
|  | Onyamboko-2014-DRC [17] | The outcomes were not relevant for this study. |
|  | Omondi-2019-KEN [18] | The outcomes were not relevant for this study. |
|  | Plucinski-2015-ANG [19] | Non-randomized trial. |
|  | Plucinski-2017-ANG [20] | Non-randomized trial. |
|  | Sylla-2013-SEN [22] | The outcomes were not relevant for this study. |
|  | Sow-2016-SEN [23] | Both children and adults were enrolled in this trial. Children’s outcome didn’t report. |
|  | Tylor-2017-UGA [24] | The outcomes were not relevant for this study. |
|  | Uwimana-2019-RWA | The outcomes were not relevant for this study. |
|  | Van-2020-MCT [25] | The outcomes were not relevant for this study. |
|  | Verret-2009-UGA [26] | The outcomes were reported in another study. |
|  | Wanzira-2014- UGA [27] | The outcomes were not relevant for this study. |
|  | Warsame-2019-SOM [28] | Both children and adults were enrolled in this trial. Children’s outcome didn’t report. |
|  | Yavo-2011-SSA [29] | Both children and adults were enrolled in this trial. Children’s outcome didn’t report. |
|  | Yeka-2013-UGA [30] | The outcomes were reported in another study. |

1. Adam I, Salah MT, Eltahir HG, Elhassan AH, Elmardi KA, Malik EM: **Dihydroartemisinin-piperaquine versus artemether-lumefantrine, in the treatment of uncomplicated Plasmodium falciparum malaria in central Sudan.** *Ann Trop Med Parasitol* 2010, **104:**319-326.

2. Borrmann S, Sasi P, Mwai L, Bashraheil M, Abdallah A, Muriithi S, et al: **Declining responsiveness of Plasmodium falciparum infections to artemisinin-based combination treatments on the Kenyan coast.** *PloS one* 2011, **6:**e26005.

3. Conrad MD, LeClair N, Arinaitwe E, Wanzira H, Kakuru A, Bigira V, et al: **Comparative impacts over 5 years of artemisinin-based combination therapies on Plasmodium falciparum polymorphisms that modulate drug sensitivity in Ugandan children.** *Journal of Infectious Diseases* 2014, **210:**344-353.

4. Creek D, Bigira V, Arinaitwe E, Wanzira H, Kakuru A, Tappero J, et al: **Increased risk of early vomiting among infants and young children treated with dihydroartemisinin-piperaquine compared with artemether-lumefantrine for uncomplicated malaria.** *Am J Trop Med Hyg* 2010, **83:**873-875.

5. Dama S, Niangaly H, Djimde M, Sagara I, Guindo CO, Zeguime A, et al: **A randomized trial of dihydroartemisinin–piperaquine versus artemether–lumefantrine for treatment of uncomplicated Plasmodium falciparum malaria in Mali.** *Malar J* 2018, **17**.

6. Davlantes E, Dimbu PR, Ferreira CM, Florinda Joao M, Pode D, Félix J, et al: **Efficacy and safety of artemether-lumefantrine, artesunate-amodiaquine, and dihydroartemisinin-piperaquine for the treatment of uncomplicated Plasmodium falciparum malaria in three provinces in Angola, 2017.** *Malaria Journal* 2018, **17**.

7. Diallo MA, Yade MS, Ndiaye YD, Diallo I, Diongue K, Sy SA, et al: **Efficacy and safety of artemisinin-based combination therapy and the implications of Pfkelch13 and Pfcoronin molecular markers in treatment failure in Senegal.** *Scientific reports* 2020, **10:**8907.

8. Ebenebe JC, Ntadom G, Ambe J, Wammanda R, Jiya N, Finomo F, et al: **Efficacy of artemisinin-based combination treatments of uncomplicated falciparum malaria in under-five-year-old Nigerian children ten years following adoption as first-line antimalarials.** *Am J Trop Med Hyg* 2018, **99:**649-664.

9. Funck-Brentano C, Bacchieri A, Valentini G, Pace S, Tommasini S, Voiriot P, et al: **Effects of Dihydroartemisinin-Piperaquine Phosphate and Artemether-Lumefantrine on QTc Interval Prolongation.** *Sci Rep* 2019, **9:**777.

10. Green JA, Mohamed K, Goyal N, Bouhired S, Hussaini A, Jones SW, et al: **Pharmacokinetic interactions between tafenoquine and dihydroartemisinin-piperaquine or artemether-lumefantrine in healthy adult subjects.** *Antimicrobial Agents and Chemotherapy* 2016, **60:**7321-7332.

11. Ishengoma DS, Mandara CI, Francis F, Talundzic E, Lucchi NW, Ngasala B, et al: **Efficacy and safety of artemether-lumefantrine for the treatment of uncomplicated malaria and prevalence of Pfk13 and Pfmdr1 polymorphisms after a decade of using artemisinin-based combination therapy in mainland Tanzania.** *Malar J* 2019, **18:**88.

12. Kakolwa MA, Mahende MK, Ishengoma DS, Mandara CI, Ngasala B, Kamugisha E, et al: **Efficacy and safety of artemisinin-based combination therapy, and molecular markers for artemisinin and piperaquine resistance in Mainland Tanzania ACTRN12615000159550 ACTRN.** *Malaria Journal* 2018, **17**.

13. Kakuru A, Jagannathan P, Arinaitwe E, Wanzira H, Muhindo M, Bigira V, et al: **The effects of ACT treatment and TS prophylaxis on Plasmodium falciparum gametocytemia in a cohort of young Ugandan children.** *American Journal of Tropical Medicine and Hygiene* 2013, **88:**736-743.

14. Katrak S, Gasasira A, Arinaitwe E, Kakuru A, Wanzira H, Bigira V, et al: **Safety and tolerability of artemether-lumefantrine versus dihydroartemisinin-piperaquine for malaria in young HIV-infected and uninfected children.** *Malaria Journal* 2009, **8**.

15. Meremikwu MM, Odey F, Donegan S, Oringanje C, Oyo-Ita A, Elemi I, et al: **Artemether-lumefantrine, artesunate+amodiaquine and dihydroartemisininpiperaquine for treating uncomplicated plasmodium falciparum malaria in under-five Nigerian children: A randomized controlled trial.** *American Journal of Tropical Medicine and Hygiene* 2013, **89:**387.

16. Muhindo MK, Kakuru A, Jagannathan P, Talisuna A, Osilo E, Orukan F, et al: **Early parasite clearance following artemisinin-based combination therapy among Ugandan children with uncomplicated Plasmodium falciparum malaria.** *Malar J* 2014, **13:**32.

17. Onyamboko MA, Fanello CI, Wongsaen K, Tarning J, Cheah PY, Tshefu KA, et al: **Randomized comparison of the efficacies and tolerabilities of three artemisinin-based combination treatments for children with acute *Plasmodium falciparum* Malaria in the Democratic Republic of the Congo.** *Antimicrob Agents Chemother* 2014, **58:**5528-5536.

18. Omondi P, Burugu M, Matoke-Muhia D, Too E, Nambati EA, Chege W, et al: **Gametocyte clearance in children, from western Kenya, with uncomplicated Plasmodium falciparum malaria after artemether-lumefantrine or dihydroartemisinin-piperaquine treatment.** *Malaria Journal* 2019, **18**.

19. Plucinski MM, Talundzic E, Morton L, Dimbu PR, Macaia AP, Fortes F, et al: **Efficacy of artemether-lumefantrine and dihydroartemisinin-piperaquine for treatment of uncomplicated malaria in children in Zaire and Uige Provinces, angola.** *Antimicrob Agents Chemother* 2015, **59:**437-443.

20. Plucinski MM, Dimbu PR, Macaia AP, Ferreira CM, Samutondo C, Quivinja J, et al: **Efficacy of artemether-lumefantrine, artesunate-amodiaquine, and dihydroartemisinin-piperaquine for treatment of uncomplicated Plasmodium falciparum malaria in Angola, 2015.** *Malaria Journal* 2017, **16**.

21. Sawa P, Shekalaghe SA, Drakeley CJ, Sutherland CJ, Mweresa CK, Baidjoe AY, et al: **Malaria transmission after artemether-lumefantrine and dihydroartemisinin-piperaquine: a randomized trial.** *J Infect Dis* 2013, **207:**1637-1645.

22. Sylla K, Abiola A, Tine RCK, Faye B, Sow D, Ndiaye JL, et al: **Monitoring the efficacy and safety of three artemisinin based-combinations therapies in Senegal: Results from two years surveillance.** *BMC Infectious Diseases* 2013, **13**.

23. Sow D, Ndiaye JL, Sylla K, Ba MS, Tine RC, Faye B, et al: **[Evaluation of the efficacy and safety of three 2-drug combinations for the treatment of uncomplicated Plasmodium falciparum malaria in Senegal: artesunate-amodiaquine, dihydroartemisinin-piperaquine, and artemether-lumefantrine].** *Med Sante Trop* 2016, **26:**45-50.

24. Taylor AR, Flegg JA, Holmes CC, Guérin PJ, Sibley CH, Conrad MD, et al: **Artemether-lumefantrine and dihydroartemisinin-piperaquine exert inverse selective pressure on plasmodium falciparum drug sensitivity-associated haplotypes in Uganda.** *Open Forum Infectious Diseases* 2017, **4**.

25. van der Pluijm RW, Tripura R, Hoglund RM, Pyae Phyo A, Lek D, ul Islam A, et al: **Triple artemisinin-based combination therapies versus artemisinin-based combination therapies for uncomplicated Plasmodium falciparum malaria: a multicentre, open-label, randomised clinical trial.** *The Lancet* 2020, **395:**1345-1360.

26. Verret WJ, Arinaitwe E, Wanzira H, Bigira V, Kakuru A, Kamya M, et al: **Effect of nutritional status on response to treatment with artemisinin-based combination therapy in young Ugandan children with malaria.** *Antimicrobial Agents and Chemotherapy* 2011, **55:**2629-2635.

27. Wanzira H, Kakuru A, Arinaitwe E, Bigira V, Muhindo MK, Conrad M, et al: **Longitudinal outcomes in a cohort of Ugandan children randomized to artemether-lumefantrine versus dihydroartemisinin-piperaquine for the treatment of malaria.** *Clin Infect Dis* 2014, **59:**509-516.

28. Warsame M, Hassan AM, Hassan AH, Jibril AM, Khim N, Arale AM, et al: **High therapeutic efficacy of artemether-lumefantrine and dihydroartemisinin-piperaquine for the treatment of uncomplicated falciparum malaria in Somalia.** *Malar J* 2019, **18:**231.

29. Yavo W, Faye B, Kuete T, Djohan V, Oga SA, Kassi RR, et al: **Multicentric assessment of the efficacy and tolerability of dihydroartemisinin-piperaquine compared to artemether-lumefantrine in the treatment of uncomplicated Plasmodium falciparum malaria in sub-Saharan Africa.** *Malar J* 2011, **10:**198.

30. Yeka A, Tibenderana J, Achan J, D'Alessandro U, Talisuna AO: **Efficacy of quinine, artemether-lumefantrine and dihydroartemisinin-piperaquine as rescue treatment for uncomplicated malaria in Ugandan children.** *PLoS One* 2013, **8:**e53772.
