# Supplementary material for: Safety of dihydroartemisinin-piperaquine versus artemether-lumefantrine for the treatment of uncomplicated Plasmodium falciparum malaria among children in Africa: a systematic review and meta-analysis of randomized control trials
Source: Malar J. 2022 Jan 4;21:4. doi: 10.1186/s12936-021-04032-2 (PMC8725395; doi:10.1186/s12936-021-04032-2)
Supplement: Supplementary file 3 — Additional file 3. Funnel plot of comparison: dihydroartemisinin-piperaquine versus artemether-lumefantrine for treatment of uncomplicated Plasmodium falciparum malaria among African children, outcome: Gastrointestinal adverse events (early vomiting). [file 12936_2021_4032_MOESM3_ESM.docx]

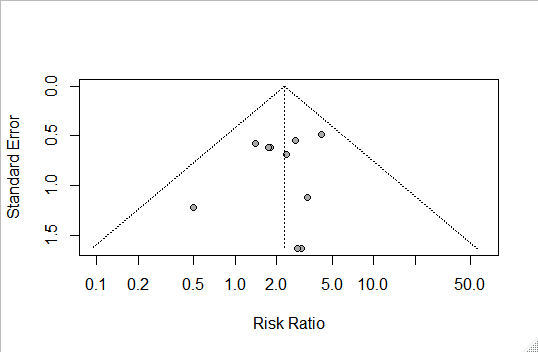


Additional file S 3: Funnel plot of comparison: dihydroartemisinin-piperaquine versus artemether-lumefantrine for treatment of uncomplicated *plasmodium falciparum* malaria among African children, outcome: Gastrointestinal adverse events (early vomiting).
