# Supplementary material for: Safety of dihydroartemisinin-piperaquine versus artemether-lumefantrine for the treatment of uncomplicated Plasmodium falciparum malaria among children in Africa: a systematic review and meta-analysis of randomized control trials
Source: Malar J. 2022 Jan 4;21:4. doi: 10.1186/s12936-021-04032-2 (PMC8725395; doi:10.1186/s12936-021-04032-2)
Supplement: Supplementary file 4 — Additional file 4. Funnel plot of comparison: dihydroartemisinin-piperaquine versus artemether-lumefantrine for treatment of uncomplicated Plasmodium falciparum malaria among African children, outcome: Gastrointestinal adverse events (diarrhoea). [file 12936_2021_4032_MOESM4_ESM.docx]

**
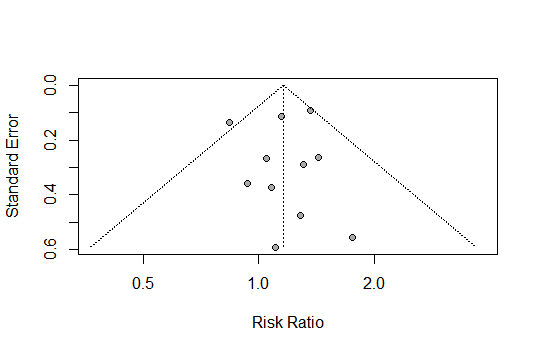
**

Additional file S 4: Funnel plot of comparison: dihydroartemisinin-piperaquine versus artemether-lumefantrine for treatment of uncomplicated *plasmodium falciparum* malaria among children in Africa, outcome: Gastrointestinal adverse events (diarrhea).
