# Supplementary material for: Safety of dihydroartemisinin-piperaquine versus artemether-lumefantrine for the treatment of uncomplicated Plasmodium falciparum malaria among children in Africa: a systematic review and meta-analysis of randomized control trials
Source: Malar J. 2022 Jan 4;21:4. doi: 10.1186/s12936-021-04032-2 (PMC8725395; doi:10.1186/s12936-021-04032-2)
Supplement: Supplementary file 5 — Additional file 5. Funnel plot of comparison: dihydroartemisinin-piperaquine versus artemether-lumefantrine for treatment of uncomplicated Plasmodium falciparum malaria among African children, outcome: Cough. [file 12936_2021_4032_MOESM5_ESM.docx]

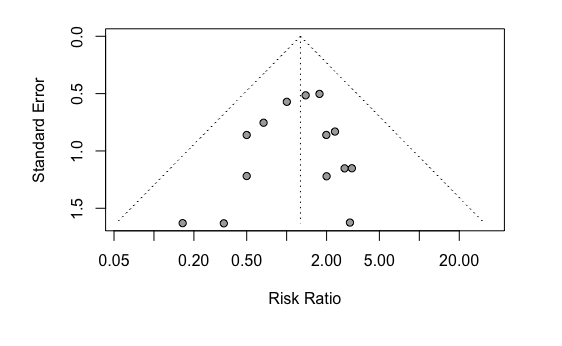


Additional file S 6: Funnel plot of comparison: dihydroartemisinin-piperaquine versus artemether-lumefantrine for treatment of uncomplicated *plasmodium falciparum* malaria among African children, outcome: Serious adverse event (including death).
