# Supplementary material for: Safety of dihydroartemisinin-piperaquine versus artemether-lumefantrine for the treatment of uncomplicated Plasmodium falciparum malaria among children in Africa: a systematic review and meta-analysis of randomized control trials
Source: Malar J. 2022 Jan 4;21:4. doi: 10.1186/s12936-021-04032-2 (PMC8725395; doi:10.1186/s12936-021-04032-2)
Supplement: Supplementary file 6 — Additional file 6. Funnel plot of comparison: dihydroartemisinin-piperaquine versus artemether-lumefantrine for treatment of uncomplicated Plasmodium falciparum malaria among African children, outcome: Serious adverse event (including death). [file 12936_2021_4032_MOESM6_ESM.docx]

| **Additional file S 7: Summary of finding table on adverse events and serious adverse event** | | | | | | |
| --- | --- | --- | --- | --- | --- | --- |
| **Dihydroartemisinin-piperaquine compared to artemether-lumefantrine for treatment of uncomplicated *plasmodium falciparum* malaria among children in Africa.** | | | | | | |
| **Patient or population: African children with uncomplicated *plasmodium falciparum* malaria**  **Setting: Malaria endemic setting in Africa.**  **Intervention: Dihydroartemisinin-piperaquine**  **Comparison: Artemether-lumefantrine** | | | | | | |
| Outcomes | **Anticipated absolute effects^*^** (95% CI) | | Relative effect (95% CI) | № of participants  (studies) | Certainty of the evidence (GRADE) | Comments |
|  | **Risk with artemether-lumefantrine** | **Risk with dihydroartemisinin-piperaquine** |  |  |  |  |
| Gastrointestinal adverse events - Early vomiting | 8 per 1,000 | **18 per 1,000** (12 to 28) | **RR 2.26** (1.46 to 3.50) | 7796 (10 RCTs) | ⨁⨁⨁⨁ HIGH ^a^ |  |
| Gastrointestinal adverse events - Diarrhea | 135 per 1,000 | **157 per 1,000** (139 to 177) | **RR 1.16** (1.03 to 1.31) | 6841 (11 RCTs) | ⨁⨁⨁⨁ HIGH ^a^ |  |
| Vomiting - Vomiting | 100 per 1,000 | **102 per 1,000** (87 to 119) | **RR 1.02** (0.87 to 1.19) | 8789 (13 RCTs) | ⨁⨁⨁⨁ HIGH ^a,c^ |  |
| Gastrointestinal Adverse event - Anorexia | 118 per 1,000 | **113 per 1,000** (100 to 127) | **RR 0.95** (0.84 to 1.07) | 6841 (11 RCTs) | ⨁⨁⨁⨁ HIGH ^a,c^ |  |
| Gastrointestinal Adverse event - Abdominal pain | 126 per 1,000 | **101 per 1,000** (72 to 140) | **RR 0.80** (0.57 to 1.11) | 2732 (8 RCTs) | ⨁⨁⨁⨁ HIGH ^a,c^ |  |
| Cardio-respiratory adverse events - Cough | 338 per 1,000 | **358 per 1,000** (341 to 375) | **RR 1.06** (1.01 to 1.11) | 8013 (13 RCTs) | ⨁⨁⨁⨁ HIGH ^a^ |  |
| Neuropsychiatry adverse event - weakness/malaise | 123 per 1,000 | **109 per 1,000** (91 to 127) | **RR 0.88** (0.74 to 1.03) | 3407 (8 RCTs) | ⨁⨁⨁⨁ HIGH ^a,c^ |  |
| Musculoskeletal/dermatological adverse events - Pruritus | 57 per 1,000 | **57 per 1,000** (32 to 101) | **RR 1.00** (0.56 to 1.78) | 1952 (5 RCTs) | ⨁⨁⨁◯ MODERATE ^a,b^ |  |
| Other Adverse events - Pyrexia | 232 per 1,000 | **218 per 1,000** (198 to 242) | **RR 0.94** (0.85 to 1.04) | 4620 (5 RCTs) | ⨁⨁⨁⨁ HIGH ^a,c^ |  |
| Serious adverse event (including death) - Serious adverse event (including death) | 8 per 1,000 | **10 per 1,000** (7 to 16) | **RR 1.27** (0.83 to 1.96) | 9558 (14 RCTs) | ⨁⨁⨁◯ MODERATE ^a,b^ |  |
| ***The risk in the intervention group** (and its 95% confidence interval) is based on the assumed risk in the comparison group and the **relative effect** of the intervention (and its 95% CI).  **CI:** Confidence interval; **RR:** Risk ratio | | | | | | |
| **GRADE Working Group grades of evidence** **High certainty:** We are very confident that the true effect lies close to that of the estimate of the effect **Moderate certainty:** We are moderately confident in the effect estimate: The true effect is likely to be close to the estimate of the effect, but there is a possibility that it is substantially different **Low certainty:** Our confidence in the effect estimate is limited: The true effect may be substantially different from the estimate of the effect **Very low certainty:** We have very little confidence in the effect estimate: The true effect is likely to be substantially different from the estimate of effect | | | | | | |

#### Explanations

a. Excluding studies with a high risk of bias didn't change the result.

b. There is no significant difference between the two treatment groups.

c. The outcome fulfilled the IOS criteria and the effect size is between 0.75 and 1.25.
